# Supplementary material for: Using surface plasmon resonance, capillary electrophoresis and diffusion-ordered NMR spectroscopy to study drug release kinetics
Source: Commun Chem. 2023 Aug 31;6:180. doi: 10.1038/s42004-023-00992-5 (PMC10471694; doi:10.1038/s42004-023-00992-5)
Supplement: Supplementary file 2 — Supplementary material [file 42004_2023_992_MOESM2_ESM.pdf]

Supporting information

Using surface plasmon resonance, capillary electrophoresis and diffusion-ordered NMR spectroscopy to study drug release kinetics

Alena Libánská[1], Tomáš Špringer[2], Lucie Peštová[2], Kevin Kotalík[1], Rafal Konefal[1], Alice Tomnikova[3], Tomáš Křížek[3], Jiří Homola[2], Eva Randárová[1], Tomáš Etrych[1]

Affiliation

[1]Institute of Macromolecular Chemistry, Czech Academy of Sciences, Prague, Czech Republic
[2]Institute of Photonics and Electronics, Czech Academy of Sciences, Prague, Czech Republic
[3]Department of Analytical Chemistry, Faculty of Science, Charles University, Prague, Czech Republic

Supplementary materials and methods

Tert-butanol (t-BuOH), dimethyl sulfoxide (DMSO), diethyl ether, ethyl acetate (EtAc), acetone, methanol (MeOH), dimethylacetamide (DMA), 2,2′-azobis(isobutyronitrile) (AIBN), 2,2′-azobis(4-methoxy-2,4-dimethylvaleronitrile) (V70), acetic acid (CH_3COOH), dexamethasone, docetaxel, 1-ethyl-3-(3-dimethylaminopropyl)-carbodiimide hydrochloride (EDC), 4-(dimethylamino)pyridine (DMAP), dimethylformamide (DMF), dichloromethane (DCM), chloroform (CCl_3), sodium borohydride ($NaBH_4$), *N*-ethylmaleimide (NEMI), cyano sodium borohydride ($NaBH_3CN$), 2,4,6-trinitrobenzenesulfonic acid (TNBSA), formic acid, tris(hydroxymethyl)aminomethane hydrochloride (Tris·HCl), acetylacetone, HABA/Avidin Reagent Kit and ethanol for spectroscopy (purity ≥ 99.9%), biotin 4-amidobenzoic acid sodium salt were obtained from Merck KGaA (Darmstadt, Germany). The 4-(2-oxopropyl) benzoic acid (OPB) and 5-methyl-4-oxo-hexanoic acid (IPL) were purchased from Rieke Metals LLC (Lincoln, Nebraska). The biotin *N*-succinimidyl ester (B-NHS) were obtained from Fluka™ (Hannover, Germany). Milli-Q water (H_2O) was used for all experiments and obtained from the Millipore appliance (Merck, Darmstadt, Germany; resistivity 18.2 MΩ/cm, 25 °C, organic carbon ≤ 5 ppb). Hexyl ester of 5-aminolevulinic acid hydrochloride (HAL) was purchased from Biosynth International, Inc. (Compton, United Kingdom). Perdeuterated dimethyl sulfoxide ($DMSO_{-d6}$) and deuterium oxide (D_2O) were purchased from Euriso-Top (Saint-Aubin, France).

N-hydroxysuccinimide (NHS) and 1-ethyl-3-(3-dimethylaminopropyl)-carbodiimide hydrochloride (EDC) for SPR were purchased from GE Healthcare (Chicago, USA). Carboxy-[HS-C_{11}-$(EG)_6$-OCH_2-COOH] (HS-OEG-COOH) and hydroxy-[HS-C_{11}-$(EG)_4$-OH] (HS-OEG-OH) oligo-ethylene glycol thiols were purchased from Prochimia (Gdynia, Poland).

Phosphate buffered saline (PBS) consisted of 1.4 mM KH_2PO_4, 8 mM Na_2HPO_4, 2.7 mM KCl and 137 mM NaCl, pH 7.4. PBS with 750 mM NaCl solution (PBS_{NaCl}) contained 1.4 mM KH_2PO_4, 8 mM Na_2HPO_4, 2.7 mM KCl and 750 mM NaCl, pH 5. Sodium acetate buffer (SA_{10}) consisted of 10 mM sodium acetate, pH 5. 2-(*N*-morpholino)ethanesulfonic acid buffer (MES_{NaCl}) was prepared from 10 mM MES with 140 mM NaCl, pH 5. Citrate buffer (CB_{NaCl}) consisted of 10 mM citrate with 140

mM NaCl, pH 3. Phosphate-citrate buffers (0.15 M) contained 45 mM Na_2HPO_4, 6 mM citric acid, pH adjusted to 7.4, or 34.2 mM Na_2HPO_4, 16.2 mM citric acid and 67 mM NaCl, pH adjusted to 5.0. All buffers were prepared using deionized water (Q-water, 18 MΩ/cm resistivity, Direct-Q UV3, Millipore, USA).

*Supplementary synthesis of the polymer precursor (**P1, P2, P3**)*

The monomers *N*-(2-hydroxypropyl)methacrylamide (HPMA) and *N*-(terc-butoxycarbonyl)-*N′*-(6-methacrylamidohexanoyl)hydrazine (Ma-Ah-NHNH-Boc) and trithiocarbonate chain transfer agent (CTA) *S*-2-cyano-2-propyl-*S′*-ethyl trithiocarbonate (sCPsE-TTC) were prepared according to the literature[1,2]. The linear copolymer precursors were prepared by controlled radical reversible addition fragmentation chain transfer (RAFT) copolymerisation of HPMA and Ma-Ah-NHNH-Boc in a molar ratio of 92:8 (**P1, P2**) and 75:25 (**P3**) using sCPsE-TTc as a CTA and V70 as an initiator. The copolymerization was performed in a mixture of t-BuOH and DMA in the volume ratio 85:15 (**P1, P2)** and 80:20 (**P3**). The molar ratio of monomers sCPsE-TTc:V70 was 500:2:1 (**P1**), 250:2:1 (**P2**) and 600:2:1 (**P3**). The copolymerisation conditions were set as previously reported[3,4] with a reaction time of 72 h for **P1 and P2** at 30°C and 20 h for **P3** at 40°C.

The trithiocarbonate ω-end groups were removed using AIBN as previously reported.[5] Briefly, **P1** (100 mg, 0.42 mmol of TTc) and AIBN (20 mg, 0.13 mmol) were dissolved in 1.2 ml of DMA (10 w/v% solution), inserted into an ampoule, bubbled with argon for 10 min and sealed. The reaction proceeded for 4 hours at 80°C. The final copolymer was precipitated into a mixture of acetone:diethyl ether (2:1), filtered and dried under a vacuum. Moreover, the additional reduction of the main-chain-end functional groups originating from CTA was performed to ensure their complete removal. Copolymer precursor **P1** (500 mg) was dissolved in dried MeOH (10 w/v% solution). $NaBH_4$ (5.5 mg, 0.15 mmol) was slowly added within 10 min followed with stirring for another 10 min, then NEMI (20 mg, 0.16 mmol) was added to the solution in two parts within 15 min. The excess of reduction agent was quenched with the addition of acetic acid (15 µl). The low molecular weight substances were separated in a LH-20 filled with MeOH with UV detection λ = 220 nm.[6] The deprotection of the hydrazide groups was performed in distilled water at 100°C as previously described.[3] The final copolymer (**P1**, **P2** or **P3**) was separated by freeze-drying.

Supplementary synthesis of drug derivate DEX and DTX

Dexamethasone 4-(2-oxopropyl) benzoate (**D-DEX**) and docetaxel 5-methyl-4-oxohexanoate (**D-DTX**) were synthesised as reported previously.[5,7] Briefly, **D-DEX** was synthesised by esterification reaction of the hydroxyl functional group on the aliphatic part of dexamethasone (DTX) and the carboxyl functional group of OPB. Similarly, the **D-DTX** was synthesised by esterification of hydroxyl group of docetaxel (DTX) and carboxyl group of IPL. Firstly, the appropriate oxo-acid (OPB or IPL) was dissolved together with the EDC in the molar ratio of 1:1.5 in the mixture of DMF and

DCM (3:1 volume ratio, 3 w/v% solution) and kept at −18°C for 20 min. Then a solution of the drug (DEX or DTX) and DMAP in molar ratio 1:1 dissolved in DMF (5 w/v% solution) was added and incubated at 4°C for 24 h. The final molar ratio of the oxo-acid:EDC:drug:DMAP was 1:1.5:1:1. The reaction course was monitored by TLC (EtAc:DCM 2:1 for DEX and EtAc for DTX). The reaction mixture was purified on a column filled with Silica gel 60 (DEX - EtAc:DCM, 8:1, with gradient lowering of the solvent ratio; DTX - EtAc; monitored by TLC). The final product was obtained after evaporation of the solvent and dried under a vacuum. HPLC showed 99 % purity (**D-DEX** peak maximum at 6.1 min, **D-DTX** peak maximum at 6.6 min) and the structures were confirmed via ^{1}H-NMR spectroscopy (**Figure SI1** and **SI2**).

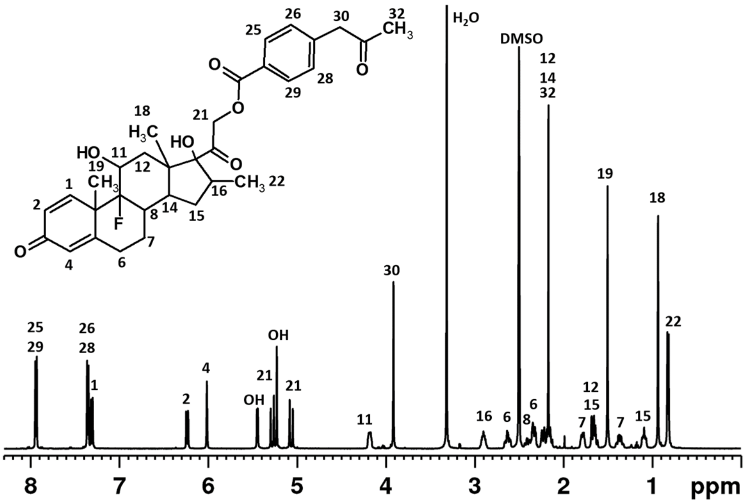

Supplementary Figure SI1: ^{1}H NMR spectrum of **D-DEX** measured in DMSO-$_{d6}$ at 22°C.

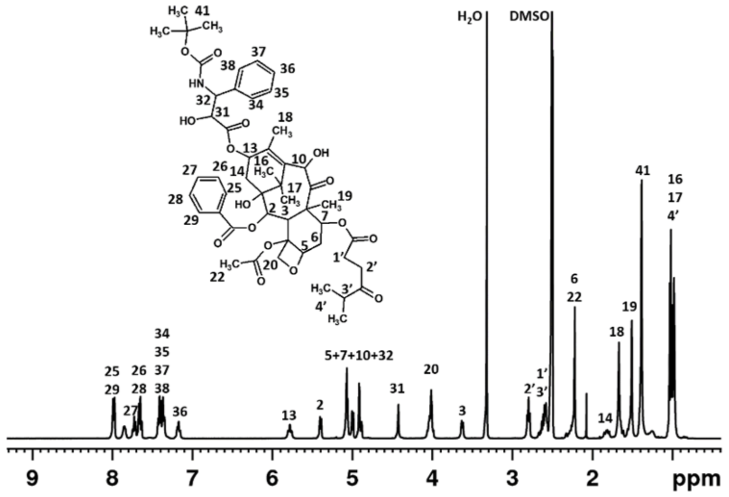

Supplementary Figure SI2: ^{1}H NMR spectrum of **D-DTX** measured in DMSO-$_{d6}$ at 22°C.

Supplementary synthesis of copolymer conjugates with biotin for SPR analysis

The copolymer conjugates with biotin were synthesised by the reaction of B-NHS with hydrazide groups of the copolymer precursors (**P1**, **P2**, **P3**) forming a physiologically stable hydrazide bond. Briefly, copolymer precursor **P1** (300 mg, 0.007 mmol) and B-NHS (20.1 mg, 0.06 mmol) were dissolved in the mixture of MeOH and DMA (8 w/v %, ratio 2:1) and stirred for 24 h at 24°C. The unbound B-NHS was removed from the polymer conjugate on a column filled with Sephadex LH-20 in MeOH with UV detection λ = 220 nm. The excess of solvent was evaporated, and the final conjugate containing bound biotin (**P1-B**) was separated by precipitation into EtAc and dried under a vacuum. The same synthetic procedure was used for the synthesis of **P2-B**, **P3-B** using **P2** or **P3** instead of **P1.**

*Supplementary synthesis of copolymer conjugates with **D-DEX** and **D-DTX***

The copolymer conjugates with the drug derivates (**P1-D-DEX, P1-B-D-DEX, P2-D-DEX**, **P2-B-D-DEX$_{5\%}$, P2-B-D-DEX$_{10\%}$, P1-D-DTX** and **P1-B-D-DTX**) were synthesised by conjugation of the appropriate drug derivate (**D-DEX** and **D-DTX**) with hydrazide groups of copolymer precursors (**P1-B**, **P2-B, P1** or **P2)** forming a pH-sensitive hydrazone bond as reported before.[4,7] Briefly, **P1** (50 mg, 0.07 mmol of $NHNH_2$ groups) and **D-DEX** (6 mg, 10 mmol) were dissolved in MeOH (10 w/v% solution) and 50 μl of CH_3COOH was added to the solution and left to react for 22 h at 10°C. The unreacted free **D-DEX** was removed on a Sephadex LH-20 column in MeOH with UV detection λ = 240 nm. The copolymer conjugate (**P1-D-DEX**) was isolated by precipitation into EtAc, filtered, and dried under a vacuum. The same synthetic procedure was used for the synthesis of **P1-B-D-DEX**, **P2-D-DEX** and **P2-B-D-DEX**, as well as for **P1-D-DTX** and **P1-B-D-DTX** using **D-DTX** instead of **D-DEX**. The weight ratio 1:0.07 of input substances (copolymer:**D-DEX**) was used for the **P2-B-D-DEX$_{5\%}$** preparation.

Supplementary synthesis of copolymer conjugates with HAL

The copolymer conjugates with HAL (**P3-HAL, P3-B-HAL)** were synthesised by conjugation of the HAL keto group with hydrazide groups of copolymer precursors (**P3**, **P3-B)** forming a pH-sensitive hydrazone bond. **P3-B** (40 mg, 0.049 mmol of $NHNH_2$ groups) or **P3** (40 mg, 0.053 mmol of $NHNH_2$ groups) was dissolved in MeOH and mixed with MeOH solution of HAL (12.32 mg, 0.049 mmol in the case of **P3-B** or 13.39 mg, 0.053 mmol in the case of **P3**) together with CH_3COOH (16 μl). The reaction mixture with a final concentration of 7 w/v% was stirred at 22°C for 24 h. The conjugates were purified from low molecular weight impurities by column chromatography (Sephadex LH-20, MeOH, UV detection λ = 220 nm). The collected fraction was concentrated under a vacuum, precipitated into EtAc and dried under a vacuum.

Supplementary characterisation of polymer precursors and conjugates

The number-average molecular weights (M_n), weight-average molecular weights (M_w), and the dispersity ($Đ$) of the polymer precursors and conjugates were measured using size-exclusion

chromatography (SEC) on an HPLC Shimadzu system equipped with an SPD-M20A photodiode array detector (Shimadzu, Japan), an OptilabrEX differential refractometer and a multi-angle light scattering DAWN HELEOS II (Wyatt Technology, Santa Barbara, CA, USA) detector using 0.15 M sodium acetate buffer at pH 6.5 (20%) and MeOH (80%, v/v) as the mobile phase for the copolymers **P1**, **P2, P1-D-DEX, P1-D-DTX, P1-B, P2-B, P1-B-D-DEX, P1-B-D-DTX, P2-B-D-DEX$_{10\%}$, P2-B-D-DEX$_{5\%}$** using a TSKgel SuperSW3000 column. The ASTRA software Version 5.3 and the refractive index increment dn/dc = 0.167 ml/g were used for calculations. For the **P3**, **P3-B** and **P3-B-HAL**, the measurements were performed on an HPLC system (Shimadzu, JP) equipped with an UV-detector, differential refractometric detector and multi-angle light scattering detector (Wyatt technology, USA) and SEC columns 1 x GRAM column 10 µm 30Å 8 x 300 mm and 2 x GRAM column 10 µm 1000Å 8 x 300 mm (PSS GmbH, Germany) connected in series using LiBr (5 mg/ml) in DMF as a mobile phase. The parameters were calculated using Astra V software and refractive index increment dn/dc = 0.1 ml/g.

The content of hydrazide groups was determined by UV/VIS spectrophotometry after derivatisation using TNBSA as previously described[3] or NMR spectroscopy (DMSO$_{-d6}$, peak at δ= 9.05 ppm)

The amount of **D-DEX** or **D-DTX** bound to the copolymers was determined after total hydrolysis of hydrazone bond by incubating conjugates for 1 h at 37°C in the mixture of water:acetonitrile 95:5 with 0.1 v/v% of TFA (pH 2) using an HPLC device equipped with a Chromolith® High Resolution RP-18e (100 x 4.6 mm) column. The amount of **D-DEX** and **D-DTX** was determined using UV detection at 240 nm. The HAL content bound to the **P3-HAL** or **P3-B-HAL** was determined using precolumn derivatisation and subsequent fluorescent detection.[8] Briefly, **P3-HAL** (1.94 mg) was dissolved in 1 ml of the mixture of water:acetonitrile 95:5 with 0.1% of TFA and incubated for 1 h at 22°C, then 20 µl of the hydrolyzed sample solution, 800 µl of first derivatization agent (15 v/v % of acetylacetone, 10 v/v % of ethanol in water) and 100 µl of second derivatization agent (10 v/v % solution of formaldehyde in water) were mixed and incubated at 100°C for 10 min. The cooled sample was analysed using an HPLC device with the fluorescent detection (excitation 370 nm, emission 460 nm).

The biotin content in all three samples **P1-B**, **P2-B** and **P3-B** was determined using a HABA/Avidin Reagent Kit (Sigma-Aldrich) according to the manufacturer's instructions and confirmed by ^{1}H-NMR analysis.

The hydrodynamic diameter (D_h) of the copolymer precursors and conjugates was measured by dynamic light scattering (DLS) using a Nano-ZS instrument (ZEN3600, Malvern) in a phosphate buffer (pH 7.4, 0.1 M). The concentration of the copolymer was 1.5 mg/ml. The intensity of the scattered light was detected at θ = 173° using a laser with a wavelength λ of 632.8 nm. The DTS (Nano) programme was used for the dynamic light scattering data evaluation. The values were extrapolated to the zero concentration and are equivalent to the mean of at least five independent measurements.

The *in vitro* drug release of **D-DEX** or **D-DTX** from **P1/P2-D-DEX** and **P1-D-DTX** were evaluated at pH 5 and pH 7.4 (0.1 M phosphate buffer) at 37°C. The concentration of the conjugate in the solution (200 µl) was 1.5 mg/ml. At predetermined time intervals, the solution was withdrawn, extracted with chloroform (800 µl), then evaporated. The released drug was dissolved in 100 µl of DMSO and analysed with an HPLC analyser (Shimadzu, Japan) equipped with diode-array detector SPD-M20A (resolution from 0.5 x 10^{-5} to 2.5 AU) using a reverse-phase column (Chromolith Performance RP-18e 100 x 4.6). The amount of **D-DEX** and **D-DTX** was determined using UV detection at 240 nm.

The structure of the drugs and the content of active compounds (**D-DEX**, **D-DTX**) bound to the copolymer precursors were determined by ^{1}H-NMR (**Figure SI3 - SI4**). The spectra were acquired using a Bruker Avance III 600 spectrometer operating at 600 MHz. The width of the ^{1}H NMR 90° pulse was 18 µs, with a relaxation delay of 10 s and an acquisition time of 2.73 s. Samples were dissolved in deuterated DMSO$_{\text{-d6}}$. The chemical shifts are relative to tetramethylsilane (TMS) using a solvent signal (DMSO, δ= 2.50 ppm from TMS in ^{1}H NMR spectra). All samples were measured at 22°C. The molar content of active compounds bound to the copolymers was calculated as follows:

$$mol\%D = \frac{I_D}{I_D + I_e}$$

where I_D is integral intensity of signal "4" for **D-DEX** (**Figure SI3**), signal "13" for **D-DTX** (**Figure SI4**) and I_e is integral intensity of signals "e" from HPMA respectively (**Figures SI3 - SI4**) and signal "1" at 6.44 ppm for biotin were used (**Figure SI6**). Similarly, the **P3-HAL** structure was determined by ^{1}H NMR (**Figure SI5**).

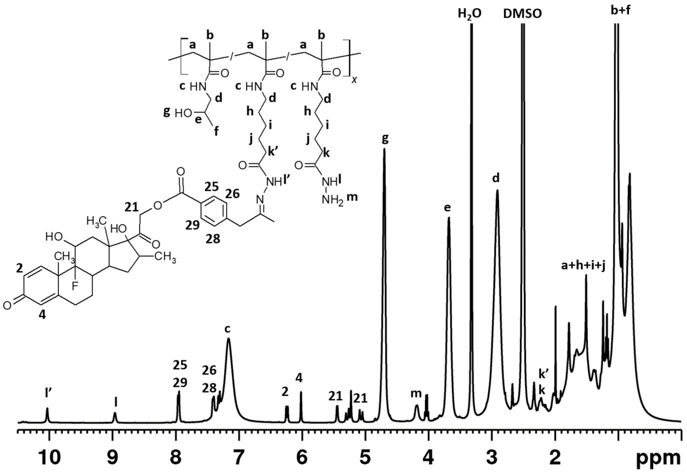

Supplementary Figure SI3:^{1}H NMR spectrum of **P1-D-DEX** measured in DMSO-$_{\text{d6}}$at 22°C.

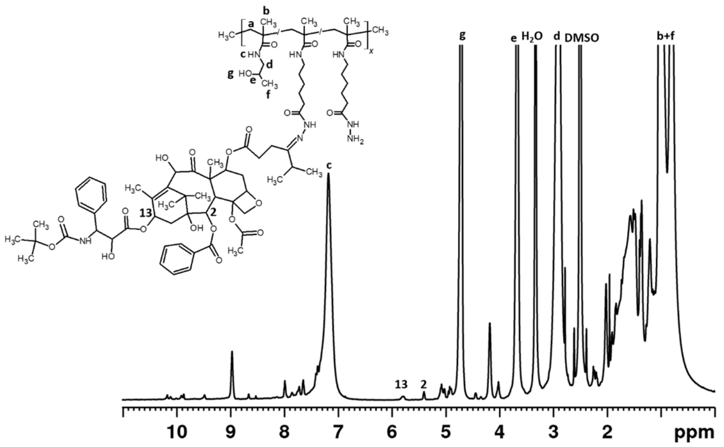

Supplementary Figure SI4: ^{1}H NMR spectrum of **P1-D-DTX** measured in DMSO-$_{d6}$ at 22°C.

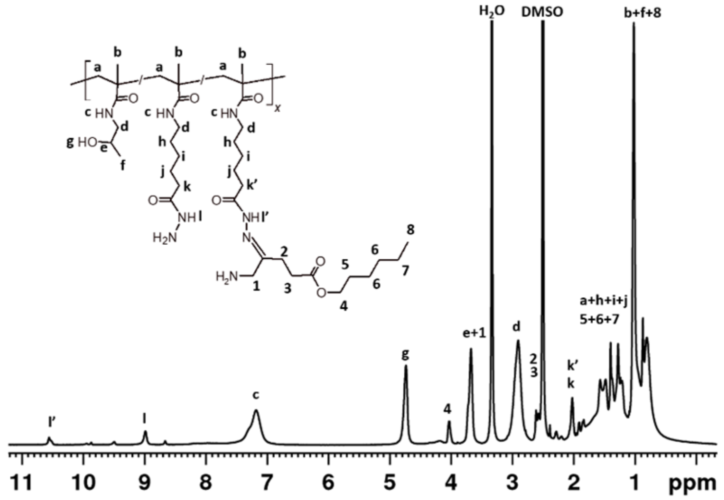

Supplementary Figure SI5: ^{1}H NMR spectrum of **P3-HAL** measured in DMSO-$_{d6}$ at 22°C.

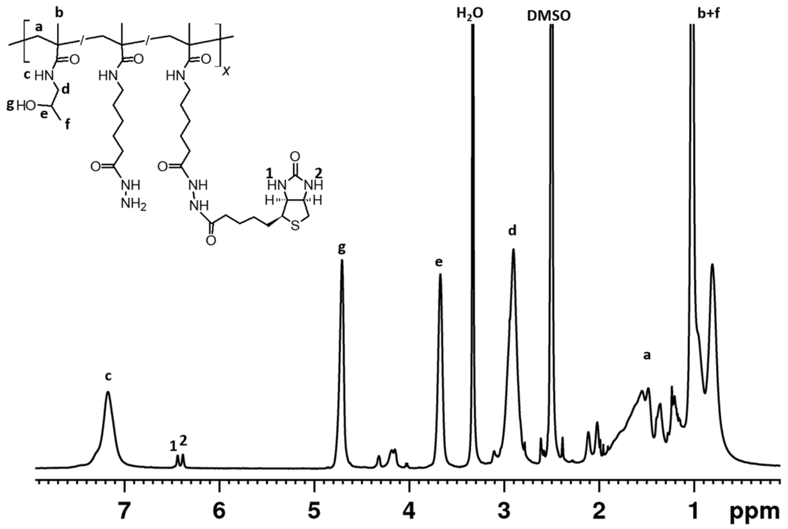

Supplementary Figure SI6: ^{1}H NMR spectrum of **P1-B** measured in DMSO-$_{d6}$ at 295 K.

Supplementary results and discussion

Supplementary application of an SPR biosensor for the drug release characterization

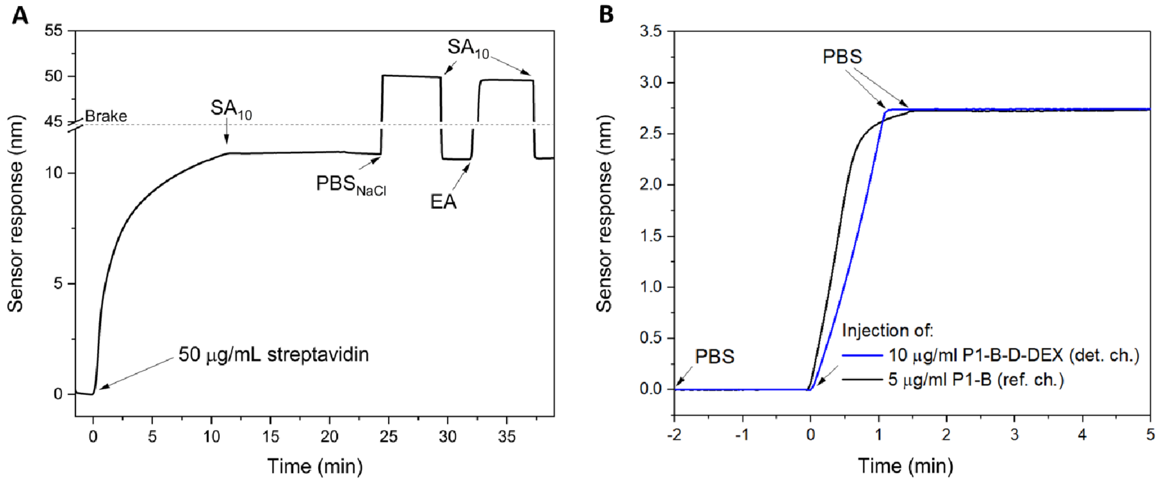

Supplementary Figure SI7: **A -** Preparation of the streptavidin-modified sensor surface of the SPR chip. **B** - SPR sensor response to binding of biotinylated **P1-B-D-DEX** and **P1-B** copolymers to streptavidin-modified sensor surfaces in the detection and reference channel, respectively.

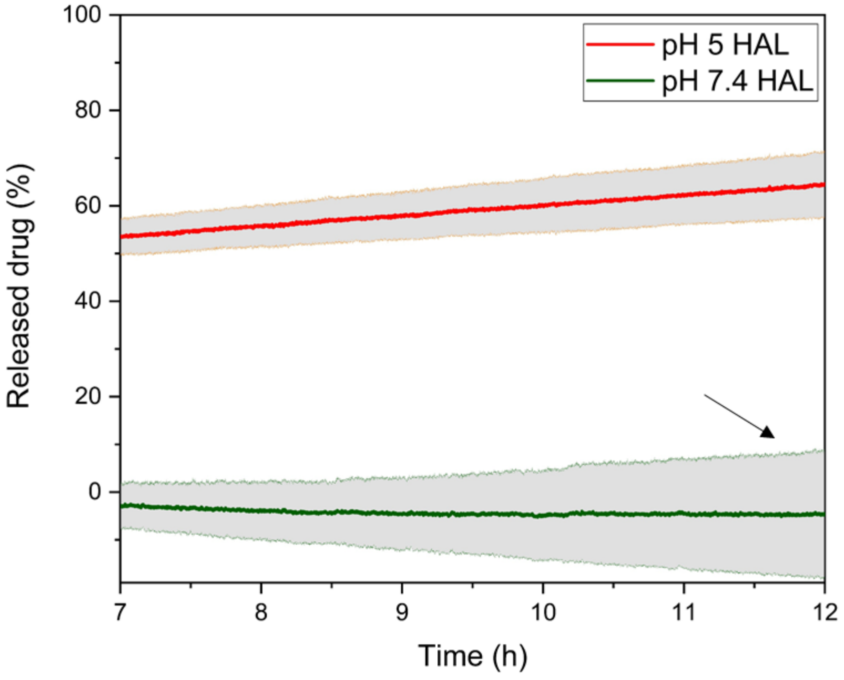

Supplementary Figure SI8: The long-term measurement of the drug release from **P3-B-HAL** at pH 7.4 with decreased measurement accuracy (black arrow) due to the interfering effects (e.g. thermal drifts, chemical instability of sensor surface).

Supplementary release kinetics determined by CE

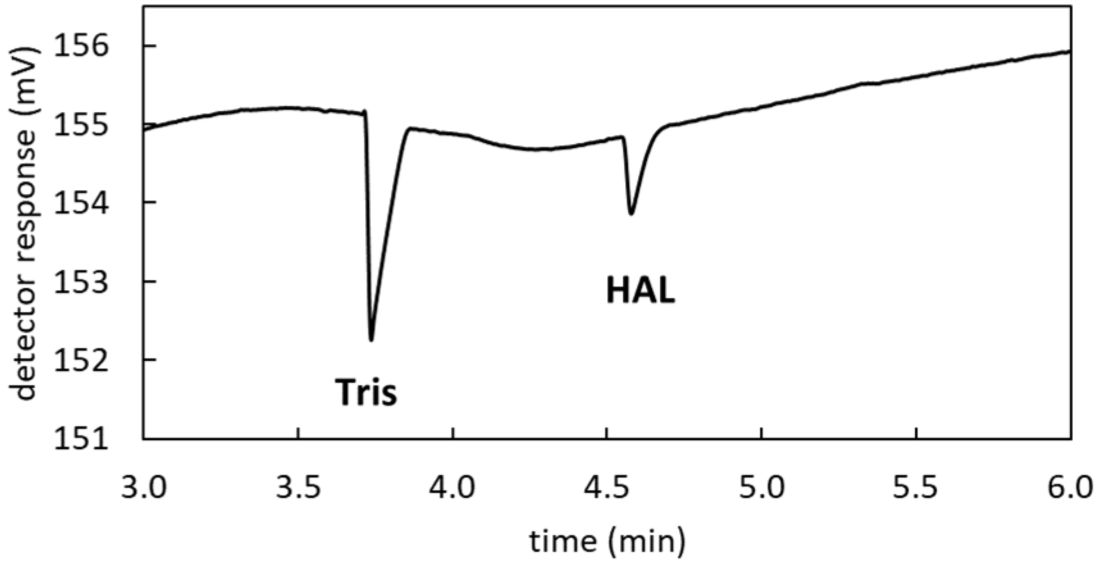

Supplementary Figure SI9: CE electropherogram of **P3-HAL** after 24 h in a phosphate-citrate buffer, pH 5.0 at 37°C. Tris cation served as an internal standard.

Supplementary release kinetics determined by NMR spectroscopy

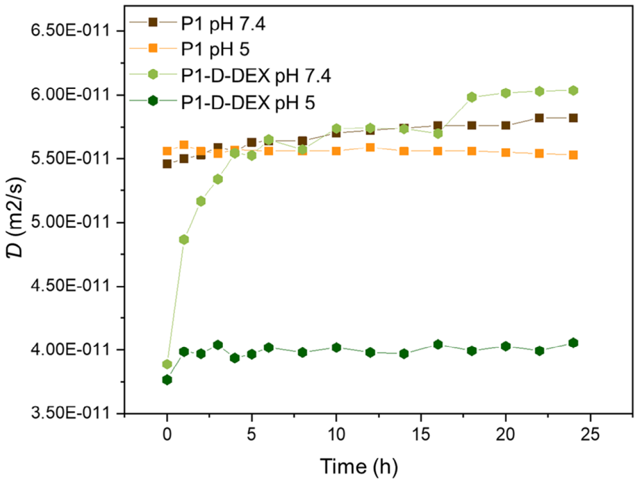

Supplementary Figure SI10: Time dependence of *D* of **P1** and **P1-D-DEX** measured over time via ^{1}H DOSY NMR at two different pHs. The relative error is ± 5 %.

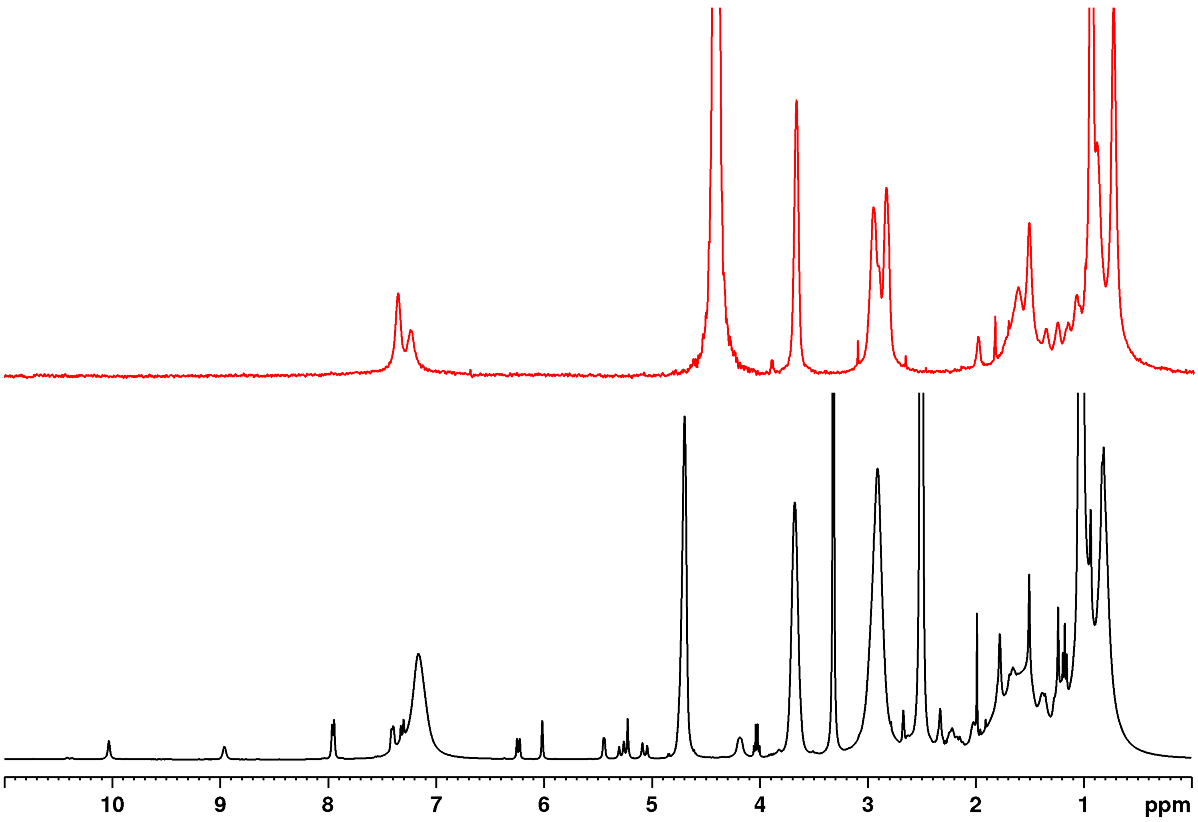

Supplementary Figure SI11: ^{1}H NMR spectra of **P1-D-DEX** measured in DMSO-$_{d6}$ at 22°C (black) and in D_2O at pH 5 at 37°C (red) at time point 0 h.

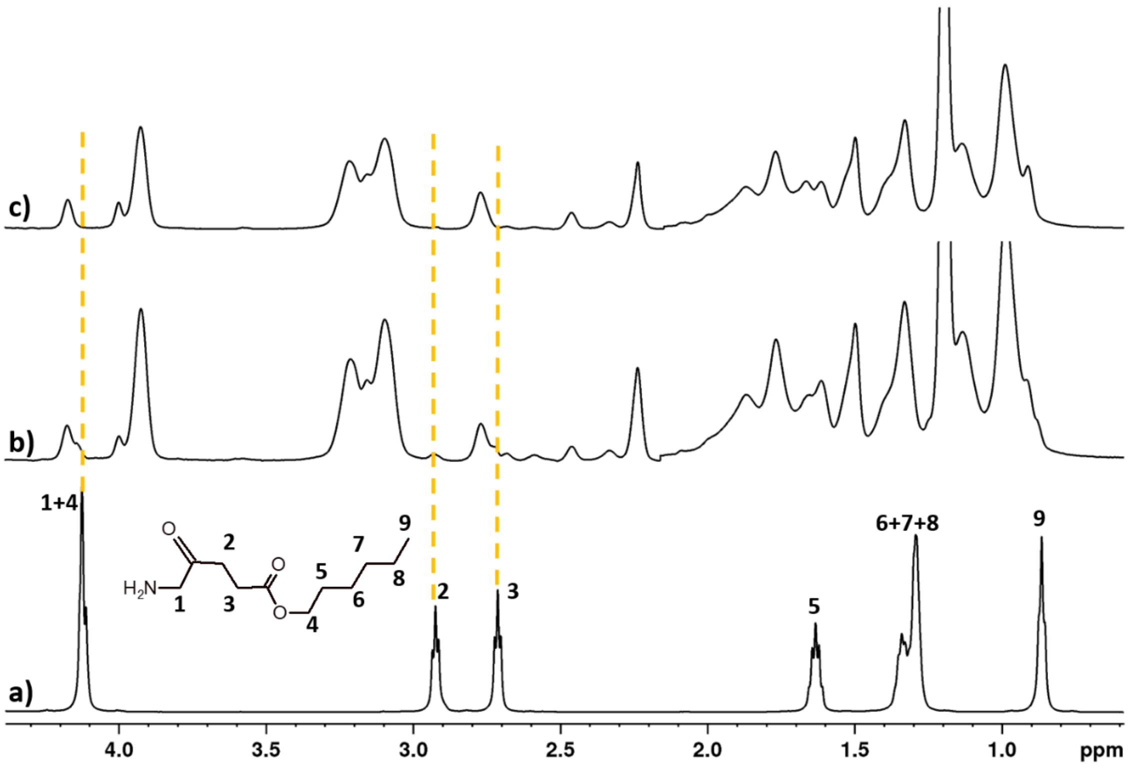

Supplementary Figure SI12: ^{1}H NMR spectra of HAL **(a), P3-HAL-24h (b) and P3-HAL-0h (c)** measured in D_2O at pH 5 at 37°C.

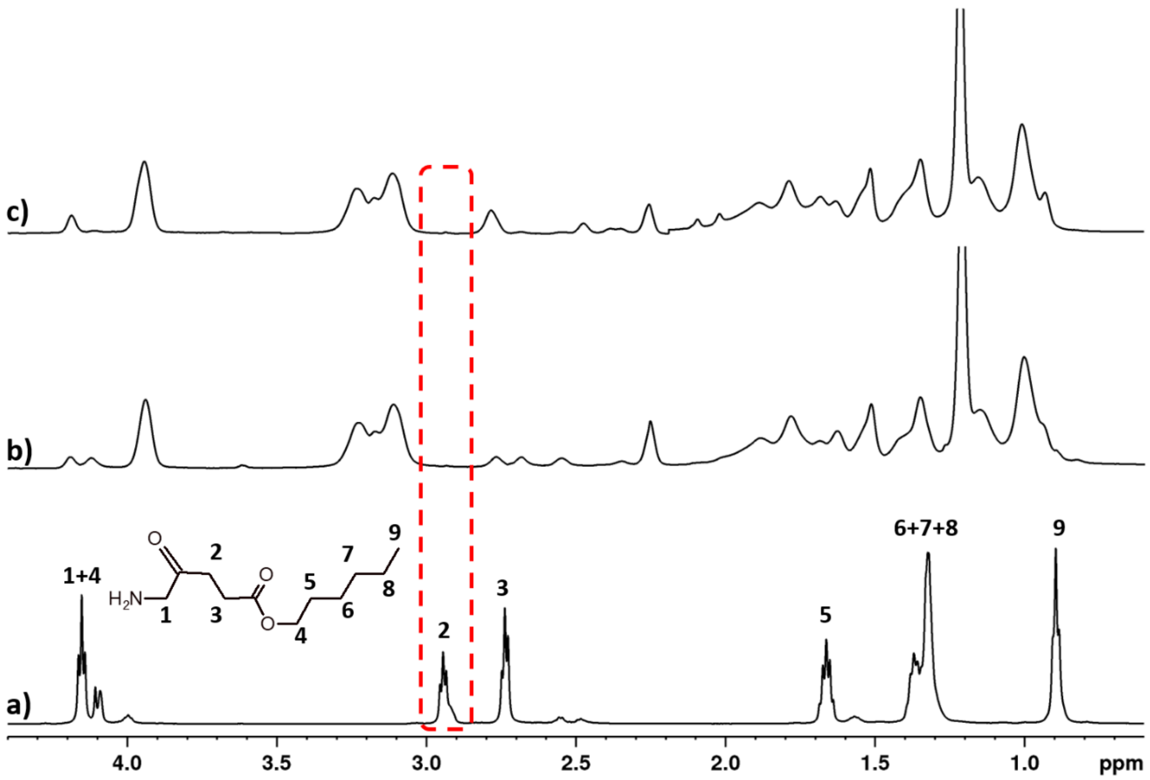

Supplementary Figure SI13: ^{1}H NMR spectra of HAL **(a), P3-HAL-24h (b)** and **P3-HAL-0h (c)** measured in D_2O at pH 7.4 at 37°C.

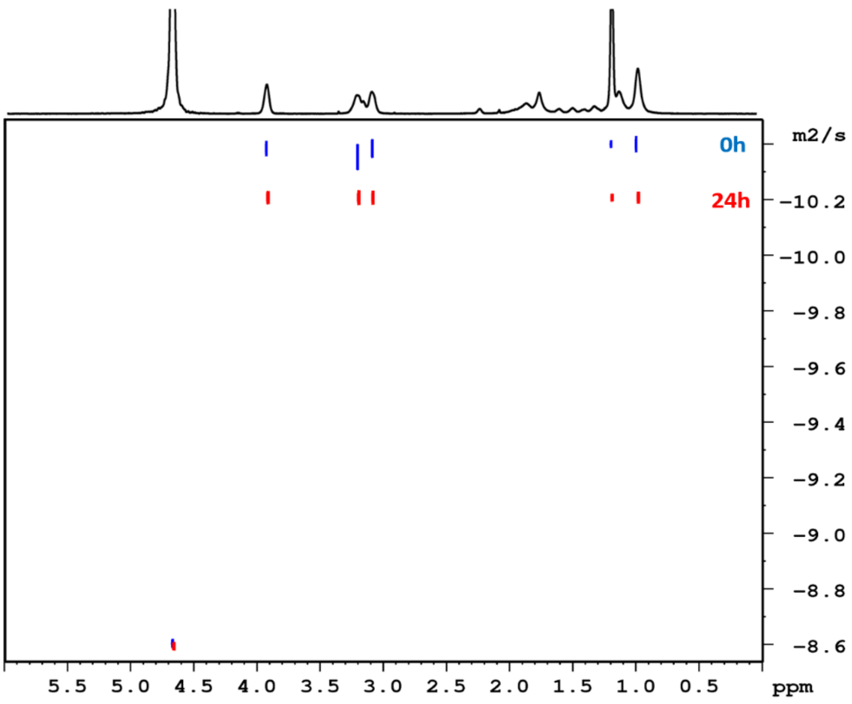

Supplementary Figure SI14: ^{1}H DOSY NMR spectra of **P1-D-DEX** measured in D_2O at pH 5 at 37°C directly after dissolving the sample (blue) and after 24 h (red).

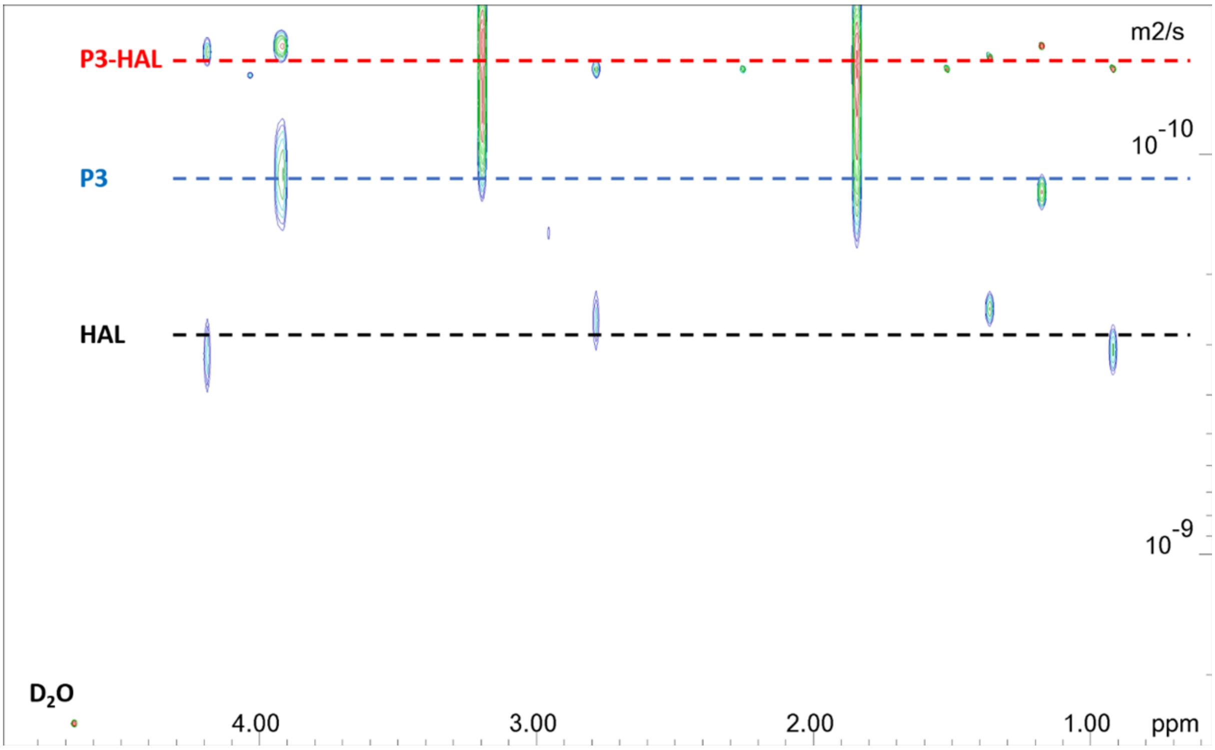

Supplementary Figure SI15: ^{1}H DOSY NMR spectrum of **P3-HAL** measured in D_2O at pH 5 at 37°C after 24 h. Two-component fitting revealed the signals related to the conjugate (**P3-HAL**), polymer precursor (**P3**) and free HAL (HAL).

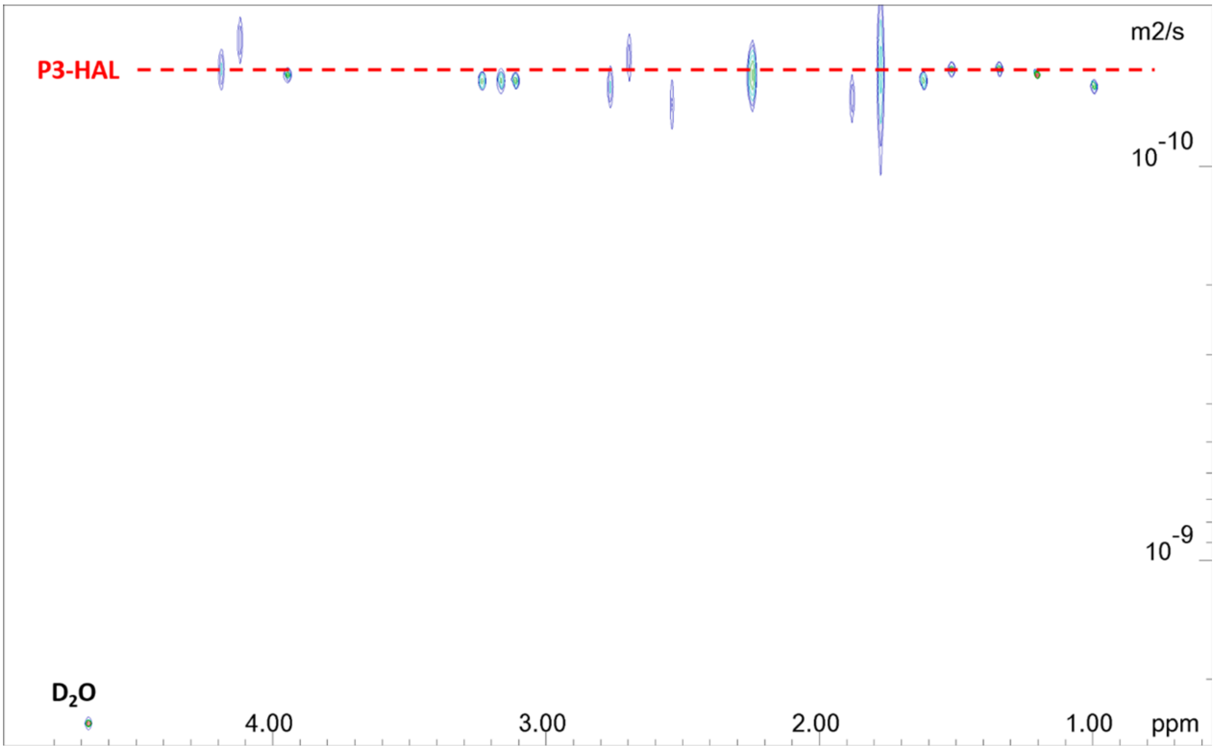

Supplementary Figure SI16: ^{1}H DOSY NMR spectrum of **P3-HAL** measured in D_2O at pH 7.4 at 37°C after 24 h. Two-component fitting only revealed the signals related to conjugate (**P3-HAL**).

Supplementary release kinetics determined by HPLC

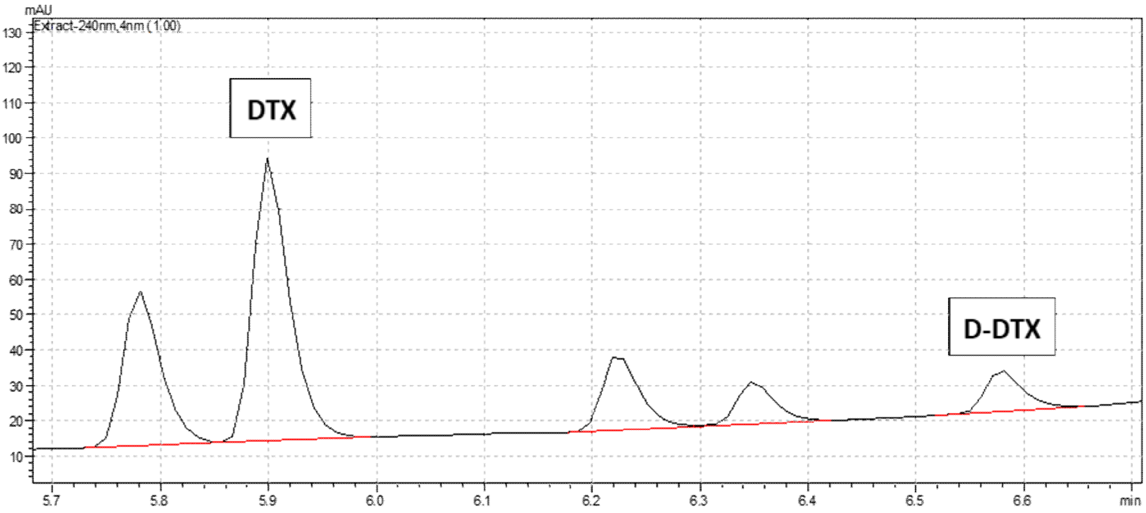

Supplementary Figure SI17: HPLC chromatogram (240 nm) of extracted products after incubation of **P1-DTX** in a phosphate buffer of pH 7.4 at 37°C.

Supplementary references:

[1] P. Chytil, T. Etrych, J. Kříž, V. Subr, K. Ulbrich, N-(2-Hydroxypropyl)methacrylamide-based polymer conjugates with pH-controlled activation of doxorubicin for cell-specific or passive tumour targeting. Synthesis by RAFT polymerisation and physicochemical characterisation., Eur. J. Pharm. Sci. Off. J. Eur. Fed. Pharm. Sci. 41 (2010) 473–482. https://doi.org/10.1016/j.ejps.2010.08.003.

[2] K. Ishitake, K. Satoh, M. Kamigaito, Y. Okamoto, Stereogradient Polymers Formed by Controlled/Living Radical Polymerization of Bulky Methacrylate Monomers, Angew. Chemie Int. Ed. 48 (2009) 1991–1994. https://doi.org/10.1002/anie.200805168.

[3] E. Koziolová, L. Kostka, L. Kotrchová, V. Šubr, R. Konefal, B. Nottelet, T. Etrych, N-(2-Hydroxypropyl)methacrylamide-Based Linear, Diblock, and Starlike Polymer Drug Carriers: Advanced Process for Their Simple Production., Biomacromolecules. 19 (2018) 4003–4013. https://doi.org/10.1021/acs.biomac.8b00973.

[4] A. Libánská, E. Randárová, F. Lager, G. Renault, D. Scherman, T. Etrych, Polymer Nanomedicines with Ph-Sensitive Release of Dexamethasone for the Localized Treatment of Inflammation, Pharm. 2020, Vol. 12, Page 700. 12 (2020) 700. https://doi.org/10.3390/PHARMACEUTICS12080700.

[5] S. Perrier, P. Takolpuckdee, C.A. Mars, Reversible Addition−Fragmentation Chain Transfer Polymerization: End Group Modification for Functionalized Polymers and Chain Transfer Agent Recovery, Macromolecules. 38 (2005) 2033–2036. https://doi.org/10.1021/ma047611m.

[6] M. Bláhová, E. Randárová, R. Konefał, B. Nottelet, T. Etrych, Graft copolymers with tunable amphiphilicity tailored for efficient dual drug delivery via encapsulation and pH-sensitive drug conjugation, Polym. Chem. 11 (2020) 4438–4453. https://doi.org/10.1039/D0PY00609B.

[7] T. Etrych, M. Sírová, L. Starovoytova, B. Ríhová, K. Ulbrich, HPMA copolymer conjugates of paclitaxel and docetaxel with pH-controlled drug release., Mol. Pharm. 7 (2010) 1015–1026. https://doi.org/10.1021/mp100119f.

[8] U. Kanto, K. Jutamanee, Y. Osotsapar, W. Chai-arree, W. Jintanawich, S. Promdang, J. Junjerm, Quantification of 5-aminolevulinic acid in swine manure extract by HPLC-fluorescence, J. Liq. Chromatogr. Relat. Technol. 36 (2013) 2731–2748. https://doi.org/10.1080/10826076.2012.725693.
