# Supplementary material for: Using surface plasmon resonance, capillary electrophoresis and diffusion-ordered NMR spectroscopy to study drug release kinetics
Source: Commun Chem. 2023 Aug 31;6:180. doi: 10.1038/s42004-023-00992-5 (PMC10471694; doi:10.1038/s42004-023-00992-5)
Supplement: Supplementary file 3 — Reporting Summary [file 42004_2023_992_MOESM3_ESM.pdf]

nature research

Corresponding author(s): DBPR, COMMSCHEM-23-0269A

Lasing Reporting Summary

Nature Research wishes to improve the reproducibility of the work that we publish. This form is intended for publication with all accepted papers reporting claims of lasing and provides structure for consistency and transparency in reporting. Some list items might not apply to an individual manuscript, but all fields must be completed for clarity.

For further information on Nature Research policies, including our data availability policy, see Authors & Referees.

Experimental design

Please check: are the following details reported in the manuscript?

1. Threshold

Plots of device output power versus pump power over a wide range of values indicating a clear threshold

☐ Yes
☒ No

Irrelevant for the study.

2. Linewidth narrowing

Plots of spectral power density for the emission at pump powers below, around, and above the lasing threshold, indicating a clear linewidth narrowing at threshold

☐ Yes
☒ No

Irrelevant for the study.

Resolution of the spectrometer used to make spectral measurements

☒ Yes
☐ No

Material and Methods section.

3. Coherent emission

Measurements of the coherence and/or polarization of the emission

☐ Yes
☒ No

Irrelevant for the study.

4. Beam spatial profile

Image and/or measurement of the spatial shape and profile of the emission, showing a well-defined beam above threshold

☐ Yes
☒ No

Irrelevant for the study.

5. Operating conditions

Description of the laser and pumping conditions
Continuous-wave, pulsed, temperature of operation

☒ Yes
☐ No

Section Materials and Method and supplementary Materials and Methods.

Threshold values provided as density values (e.g. W cm^{-2} or J cm^{-2}) taking into account the area of the device

☐ Yes
☒ No

Irrelevant for the study.

6. Alternative explanations

Reasoning as to why alternative explanations have been ruled out as responsible for the emission characteristics
e.g. amplified spontaneous, directional scattering; modification of fluorescence spectrum by the cavity

☐ Yes
☒ No

No alternative explanations were ruled out.

7. Theoretical analysis

Theoretical analysis that ensures that the experimental values measured are realistic and reasonable
e.g. laser threshold, linewidth, cavity gain-loss, efficiency

☐ Yes
☒ No

The article is dealing with comparison of three analytical method, thus the theoretical analysis was not necessary.

8. Statistics

Number of devices fabricated and tested

☐ Yes
☒ No

We did not fabricated any device.

Statistical analysis of the device performance and lifetime (time to failure)

☐ Yes
☒ No

We did not fabricated any device.

March 2018
